# Development and characterisation of interspecific hybrid lines with genome-wide introgressions from *Triticum timopheevii* in a hexaploid wheat background

**DOI:** 10.1186/s12870-019-1785-z

**Published:** 2019-05-06

**Authors:** Urmila Devi, Surbhi Grewal, Cai-yun Yang, Stella Hubbart-Edwards, Duncan Scholefield, Stephen Ashling, Amanda Burridge, Ian P King, Julie King

**Affiliations:** 1 Division of Plant and Cop Sciences, The University of Nottingham, Sutton Bonington Campus, Loughborough, Leicestershire UK; 2 Cereal Genomics Lab, Life Sciences Building, School of Biological Sciences, University of Bristol, Bristol, UK

**Keywords:** Wheat, *Triticum timopheevii*, Introgression, Interspecific hybrid, SNP, Genetic mapping, Wild relatives

## Abstract

**Background:**

*Triticum timopheevii* (2n = 4x = 28; A^t^A^t^GG), is an important source for new genetic variation for wheat improvement with genes for potential disease resistance and salt tolerance. By generating a range of interspecific hybrid lines, *T. timopheevii* can contribute to wheat’s narrow gene-pool and be practically utilised in wheat breeding programmes. Previous studies that have generated such introgression lines between wheat and its wild relatives have been unable to use high-throughput methods to detect the presence of wild relative segments in such lines.

**Results:**

A whole genome introgression approach, exploiting homoeologous recombination in the absence of the *Ph1* locus, has resulted in the transfer of different chromosome segments from both the A^t^ and G genomes of *T. timopheevii* into wheat. These introgressions have been detected and characterised using single nucleotide polymorphism (SNP) markers present on a high-throughput Axiom® Genotyping Array. The analysis of these interspecific hybrid lines has resulted in the detection of 276 putative unique introgressions from *T. timopheevii*, thereby allowing the generation of a genetic map of *T. timopheevii* containing 1582 SNP markers, spread across 14 linkage groups representing each of the seven chromosomes of the A^t^ and G genomes of *T. timopheevii*. The genotyping of the hybrid lines was validated through fluorescence in situ hybridisation (FISH). Comparative analysis of the genetic map of *T. timopheevii* and the physical map of the hexaploid wheat genome showed that synteny between the two species is highly conserved at the macro-level and confirmed the presence of inter- and intra-genomic translocations within the A^t^ and G genomes of *T. timopheevii* that have been previously only detected through cytological techniques.

**Conclusions:**

In this work, we report a set of SNP markers present on a high-throughput genotyping array, able to detect the presence of *T. timopheevii* in a hexaploid wheat background making it a potentially valuable tool for marker assisted selection (MAS) in wheat pre-breeding programs. These valuable resources of high-density molecular markers and wheat-*T. timopheevii* hybrid lines will greatly enhance the work being undertaken for wheat improvement through wild relative introgressions.

**Electronic supplementary material:**

The online version of this article (10.1186/s12870-019-1785-z) contains supplementary material, which is available to authorized users.

## Background

Wheat yields are plateauing in many countries at a time when production needs to be increased to feed the ever-growing population [3, 7]. A key factor in the plateauing of yields observed is the relatively small amount of genetic variation available in the gene pool of hexaploid wheat that can be used to develop new superior high yielding varieties adapted to the changing environment. However, unlike wheat its wild relatives provide a vast and almost untapped source of genetic variation that could be exploited to provide a step change in wheat breeding programmes (for reviews see [16, 29, 54]).

*Triticum timopheevii* is a tetraploid (2n = 4x = 28; A^t^A^t^GG) member of the triticeae. An allopolyploid, the progenitor species are thought to be the same as those for *Triticum turgidum* and *Triticum aestivum*, with *Triticum urartu* contributing the A^t^ genome [11] and an *Aegilops speltoides*-like species the G genome [12, 44]. A study on chromosome pairing by Rodriguez et al. [52] suggested that the G genome of *T. timopheevii* and the S genome of *Ae. speltoides* are closer together than either are to the B genome of *T. aestivum*. Meiotic analysis by Feldman [14] of F_1_ hybrids between *T. timopheevii* and *T. aestivum* showed that the chromosomes of the B and G genomes paired to form bivalents, etc., only 30% of the time. In contrast the A and A^t^ genomes, appear to be more closely related as in the same study by Feldman [14] the chromosomes from these genomes paired 70% of the time.

It is thought that the timopheevii wheats (including *T. timopheevii*) arose from a separate hybridisation event to *T. turgidum* and *T. aestivum*, based on the presence of different species-specific translocations. *T. timopheevii* contains the 4A^t^L/5A^t^L translocation found in *T. turgidum* and *T. aestivum* but also a 6A^t^/1G/4G cyclic translocation [23]. However, like wheat, *T. timopheevii* carries a pairing control locus located on chromosome 5G, which acts to suppress homoeologous recombination. Chromosome 5B of wheat carries the *Ph1* locus which also restricts pairing to homologous chromosomes. However, although this indicates a possible relationship, the *Ph1* locus in wheat is thought to be stronger than that found on chromosome 5G of *T. timopheevii* [45].

*T. timopheevii* has been shown to be a valuable source of new disease resistance genes including leaf rust resistance [5, 31, 57, 59], stem rust resistance [1, 26, 47, 48, 63], powdery mildew resistance [21, 24, 49] and Fusarium head blast resistance [4, 6, 13]. In addition to the resistance genes, *T. timopheevii* has been shown to contain genetic variation for salt tolerance [67] and protein content [43, 69].

One of the most effective ways of introducing new genetic variation from wild relatives into wheat is by the generation of introgressions via homoeologous recombination and indeed introgressions previously produced between *T. aestivum* and *T. timopheevii* have shown the potential value of this approach [2, 17, 51]. A major bottleneck for the introduction of genetic variation into wheat from its wild relatives has been the difficulty in detecting and characterising introgressions. However, King et al. [27] reported the development of an Axiom® array, composed of circa 35 K single nucleotide polymorphism (SNP) markers, able to detect polymorphisms between wheat and 10 wild relatives including *T. timopheevii*. To date, this array has enabled the large-scale detection and characterisation of introgressions in wheat from *Ambylopyrum muticum*, *Ae. speltoides*, *Thinopyrum bessarabicum* and *T. urartu* [18, 19, 27, 28].

This paper reports the development of genome wide introgressions from *T. timopheevii* into wheat. At the Nottingham/BBSRC Wheat Research Centre (WRC) the Axiom® Wheat-Relative Genotyping Array was used to both detect and characterise the introgression lines produced, via the generation of a genetic linkage map of *T. timopheevii* consisting of 1582 SNP markers spread across both the A^t^ and G genomes. The genetic linkage map was also validated using fluorescence in situ hybridisation (FISH).

## Results

### Generation of wheat-*T. timopheevii* introgressions through homoeologous recombination in the absence of the *Ph1* locus

**Fig. 1:**
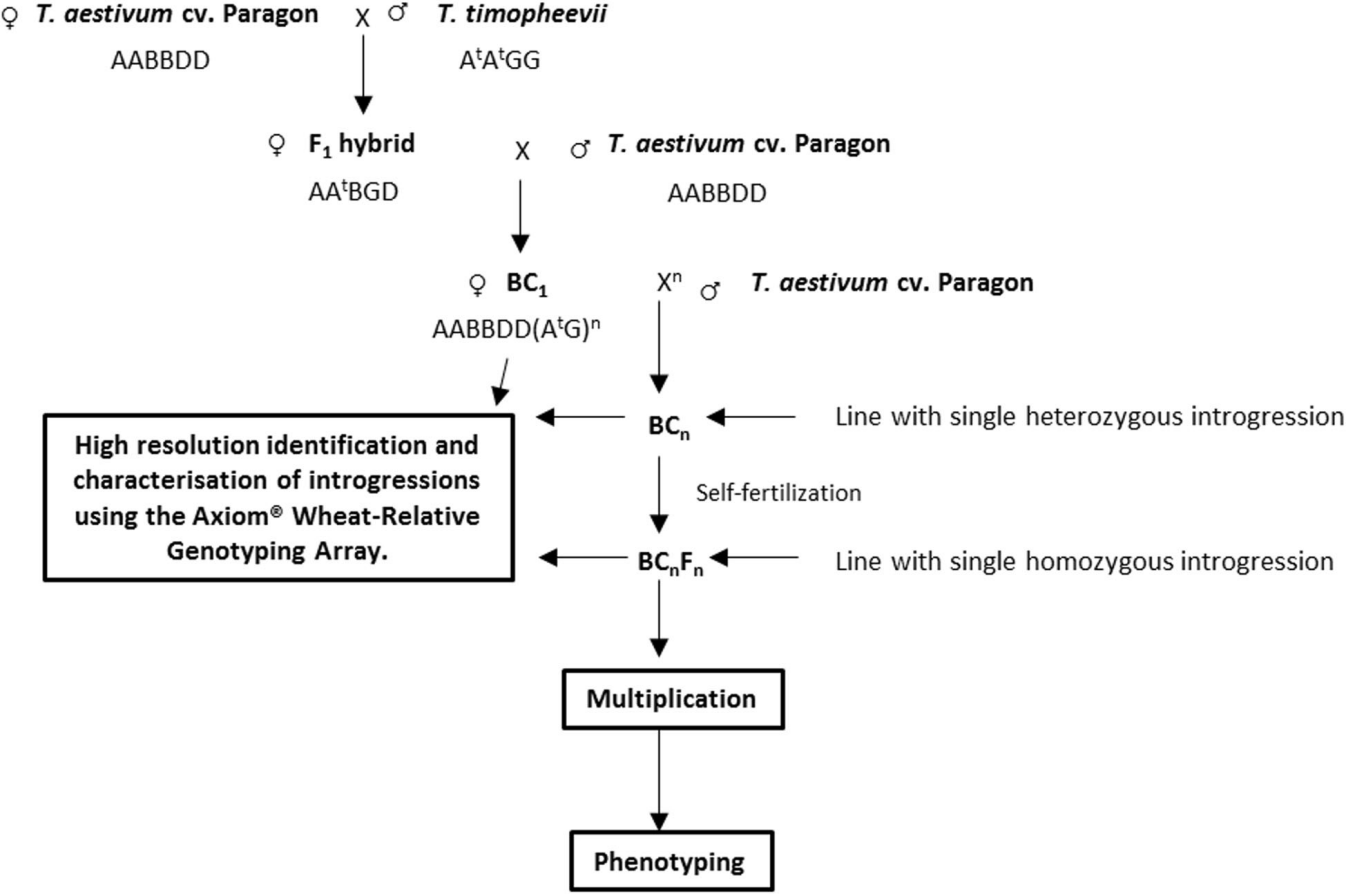
Wheat/*T. timopheevii* crossing programme undertaken in this work

In order to generate wheat-*T. timopheevii* introgressions lines (Fig. 1) a total of 1947 crosses were made leading to the generation of 12,883 crossed seed and 7018 self-seed. The number of crosses made and seed set in each generation is shown in Table 1. In total, 150 interspecific F_1_ seeds were generated by crossing *T. timopheevii* with wheat having a mutation at the *Ph1* locus resulting in homoeologous recombination between the chromosomes of the two species. Of these F_1_ hybrids, 86 were selected at random to produce the subsequent generations. Only 73 of these plants germinated, of which 25 (34%) produced seed when backcrossed to wild type Paragon in order to produce the BC_1_ generation. In contrast, the number of plants (again derived from randomly selected seed) that set seed in the BC_1_, BC_2_, BC_3_ and BC_4_ generations was close to or at 100%.

**Table 1:** Number of seeds produced and germinated in relation to the number of crosses carried out, cross fertility and the number of self-fertilised seed produced for each generation of the introgression programme for *T. timopheevii* into hexaploid wheat

	Seeds sown	Germination rate (%)	Crosses made	Cross fertility (%)	Crossed seeds produced	Seeds/Cross	Self-fertilised seeds produced
Wheat *× T. timopheevii*	–	–	424	5	150	0.3	–
F_1_	86	85	351	16	152	0.4	0
BC_1_	90	42	204	94	1317	6.5	395
BC_2_	187	78	641	93	6398	10	3077
BC_3_	101	81	312	99	4637	15	3471
BC_4_	6	67	15	100	229	15.3	75
Total	470	**–**	1947	**–**	12,883	**–**	7018

The lowest frequencies of fertility were observed in the crosses between Paragon *ph 1/ph1* x *T. timopheevii* and between the F_1_ x wild type Paragon with only 5 and 16% of crossed ears setting seed respectively. In contrast, the BC_1_, BC_2_, BC_3_ and BC_4_ generations showed much higher levels of fertility with 94, 93, 99 and 100% of crossed ears setting seed respectively. In addition, the average number of seed set per crossed ear was also considerably lower in the interspecific cross between Paragon *ph 1/ph1* x *T. timopheevii* and the F_1_ x wild type, i.e. 0.3 and 0.4% respectively as compared to the BC_1_, BC_2_, BC_3_ and BC_4_ generations, i.e. 6.5, 10, 15 and 15% respectively. Self-fertilised seed was obtained from each generation with the exception of the F_1_.

### Detection of introgressions using the Axiom^®^ Wheat-Relative Genotyping Array

To detect introgressions from *T. timopheevii* in the interspecific hybrid lines, a 35 K Axiom^®^ Wheat-Relative Genotyping Array was used. Of the SNPs on the array, 19,460 showed polymorphism between Paragon and *T. timopheevii* (Table 2). DNA from 344 individuals of BC_1_-BC_**4**_ populations were screened with the array. Genotype calls were generated, and the sample call rate ranged from 88.8 to 99.9% with an average of 99%. The lowest call rates were obtained for the three *T. timopheevii* samples with an average of 89.6%. The scores for each SNP was classified into one of six cluster patterns using Affymetrix software. Only those classified as Poly High Resolution (PHR; 3256) and thus, considered to be of optimum quality were used for genetic mapping. A majority of the polymorphic SNPs (10,537) were classified under the 'No minor homozygous allele' category. These were not considered to be ideal for genotyping a segregating backcross population due to the presence of heterozygous alleles, in at least one parent, for these SNPs.

**Table 2:** Number of SNP markers polymorphic between wheat and *T. timopheevii* on the Axiom® Wheat-Relative Genotyping Array and final number of SNP markers mapped onto the genetic map of the A^t^ and G genomes of *T. timopheevii*, and corresponding genetic distances (in cM) of each linkage group in the genetic maps obtained through Poly High Resolution (PHR) calling

	SNP markers on Array	% of Total SNP markers	PHR calls on genetic map of A^t^ genome	cM length	PHR calls on genetic map of G genome	cM length
Linkage Group 1	2521	13.0	66	46.8	155	16.6
Linkage Group 2	3567	18.3	70	45.8	137	76.5
Linkage Group 3	2902	14.9	90	79.2	77	38.4
Linkage Group 4	2364	12.1	37	15.3	121	43.2
Linkage Group 5	3063	15.7	99	90.8	218	50.7
Linkage Group 6	2198	11.3	54	52.0	175	41.6
Linkage Group 7	2845	14.6	68	54.6	161	23.7
Total	19,460	100.00	484	384.5	1044	290.7

### Genetic mapping of *T. timopheevii* chromosomes

**Fig. 2:**
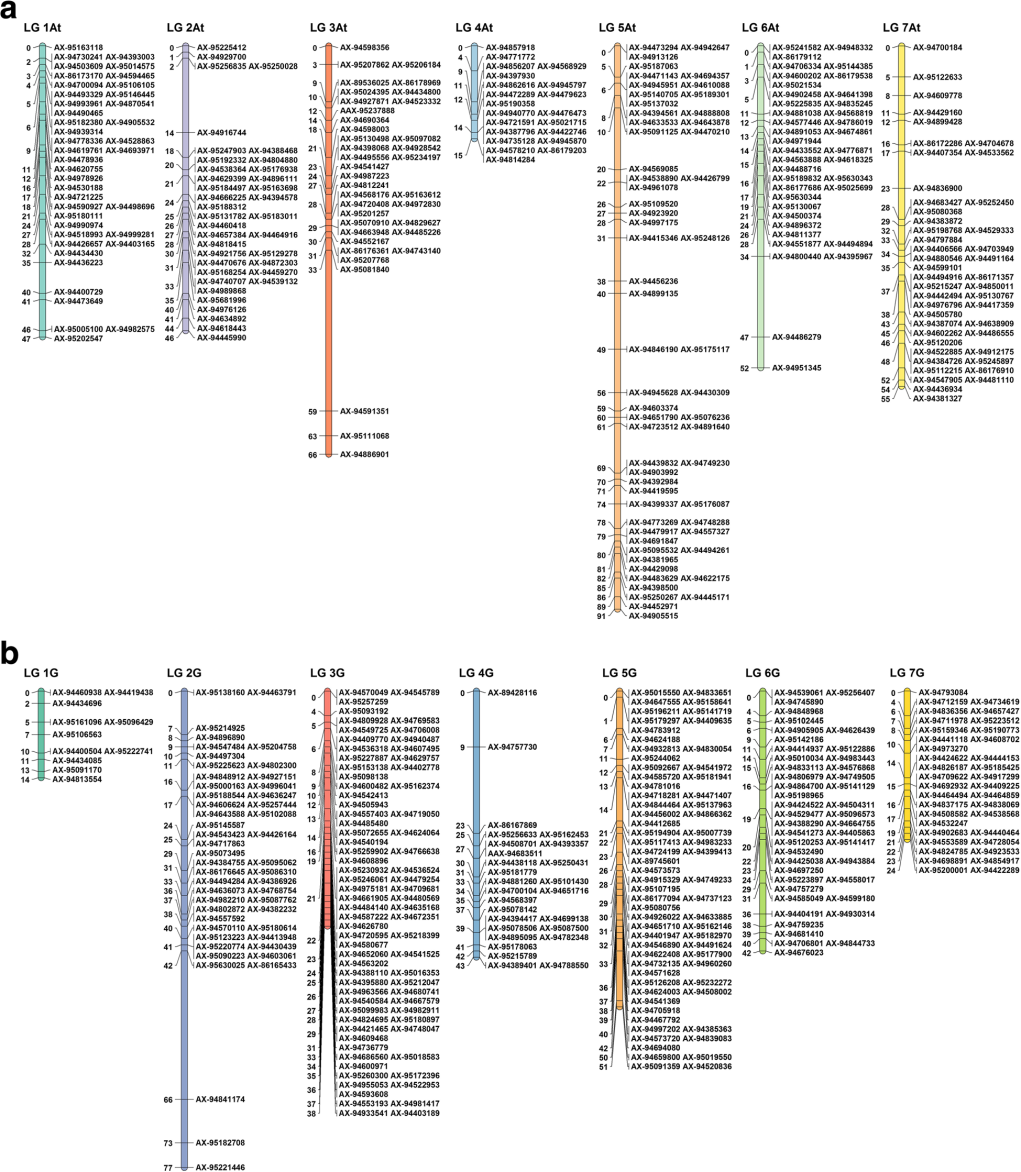
Genetic linkage map of (**a**) *T. timopheevii* A^t^ genome (**b**) *T. timopheevii* G genome

Joinmap [61] was used to analyse the PHR SNPS which resulted in the generation of 14 linkage groups with a total of 1528 SNPs. Each linkage group was assigned to either the A^t^ or the G genome of *T. timopheevii* depending on the BLAST analysis of the markers against the wheat genome. If the linkage group had most top hits in the A genome of wheat, it was assigned to the A^t^ genome of *T. timopheevii*. In contrast, if most top hits in the BLAST analysis were from the B genome of wheat then the linkage group was assigned to the G genome of *T. timopheevii*. Assuming synteny between wheat and *T. timopheevii*, the linkage groups were assigned to the same homoeologous group in the latter as indicated by the BLAST results in wheat, i.e. markers which produced a top hit on chromosome group 5 in wheat were assigned to linkage group 5 in *T. timopheevii*. The genetic map of the A^t^ genome of *T. timopheevii* was composed of 484 SNPs (Fig. 2a) and that of the G genome was composed of 1044 SNPs (Fig. 2b). The number of SNPs assigned to each of the 7 linkage groups of the A^t^ and G genomes varied between groups as is shown in Table 2. In both the A^t^ and G genomes, linkage group 5 had the highest number of SNPs (linkage group 5A^t^ = 20.5% and linkage group 5G = 20.9% of the SNPs on the genetic maps of the A^t^ and G genomes respectively) and linkage group 4 had the lowest number (linkage group 4A^t^ = 7.6% and linkage group 4G = 11.6% of the SNPs on the genetic maps of the A^t^ and G genomes respectively). The total length of the genetic map of the A^t^ genome was 384.5 cM and the total length of the map of the G genome was 290.7 cM. The length in cM of each linkage group varied considerably. For example, linkage group 4 of the A^t^ genome was only 15.3 cM in contrast to linkage group 5 which was 90.8 cM. For the G genome, linkage group 1 was 16.6 cM in contrast to linkage group 2 which was 76.5 cM (Table 2).

**Fig. 3:**
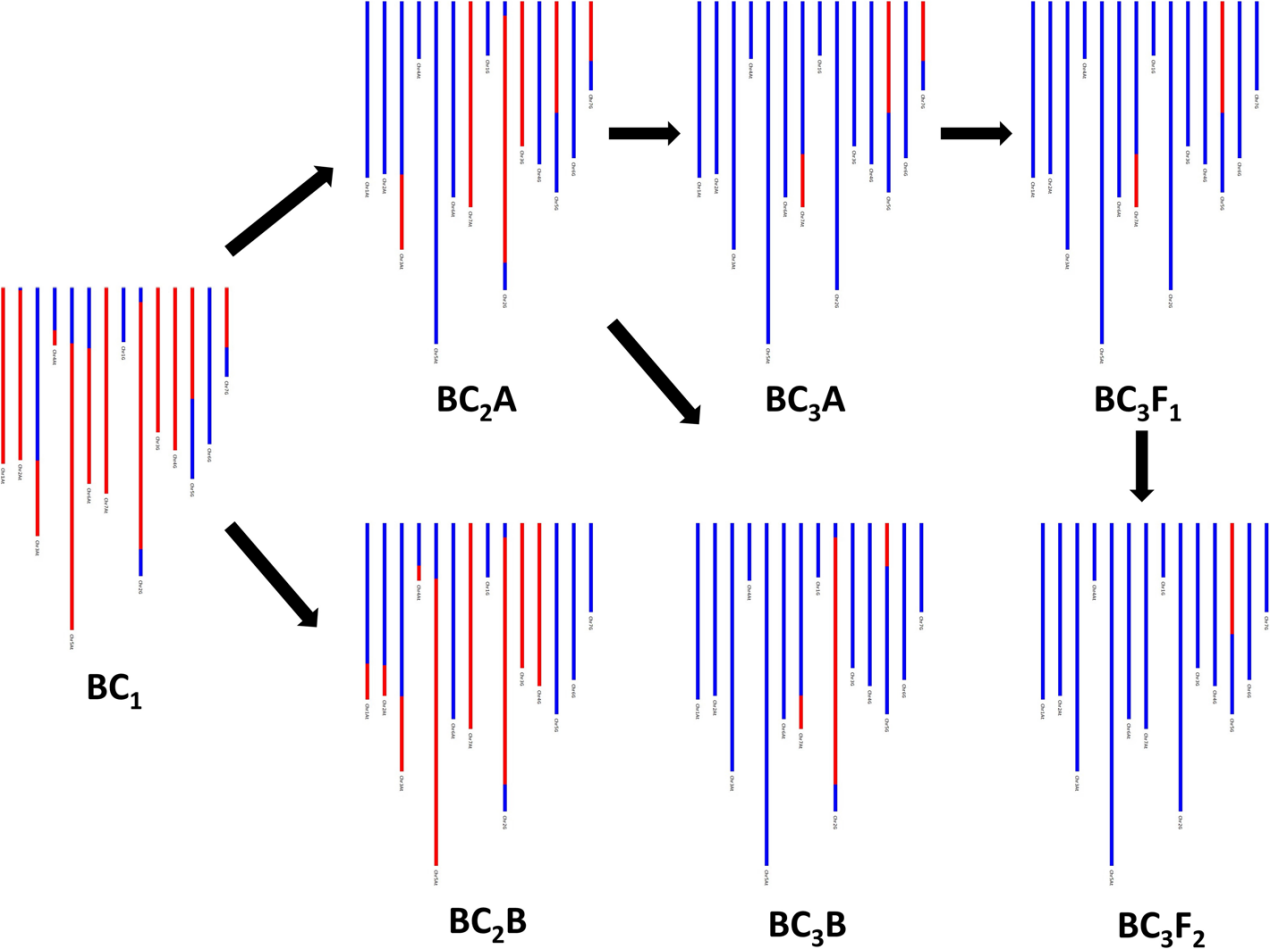
Marker assisted selection of *T. timopheevii* introgressions across a family of back-cross lines. The red colour, in the GGT bar diagrams, is used to represent the presence of a *T. timopheevii* introgression, while the blue colour represents wheat (these diagrams cannot be used to assess which wheat chromosomes the *T. timopheevii* segments have recombined with). Recombination can be seen to have occurred in linkage group 1A^t^ between the BC_1_ and BC_2_ generations. Eventually the markers allow selection of a BC_3_F_2_ line containing a single *T. timopheevii* segment from linkage group 5G

Genotyping indicated that, in total, 276 potential introgressions had been generated between wheat and *T. timopheevii*; 141 between wheat and the A^t^ genome and 135 between wheat and the G genome (Fig. 2). Marker analysis revealed that recombination between the genomes of wheat and those of *T. timopheevii* was not restricted to the gametes of the F_1_, i.e. recombination between the genomes of the two species also occurred during gametogenesis in the backcross progenies, e.g. linkage group 1A^t^ shows recombination between BC_1_ and BC_2_B and linkage group 5G shows recombination between BC_2_A and BC_3_B (Fig. 3).

The number of introgressions retained decreased considerably in each backcross population with one exception. For example, 71% of the BC_1_ genotypes carried introgressions from linkage group 1 of the A^t^ genome as compared to 50, 29 and 21% in the BC_2_, BC_3_ and BC_4_ generations respectively (Table 3). However, in contrast, linkage group 2 from the G genome which was present in 94% of the BC_1_ genotypes was still present in 75, 74 and 70% in the BC_2_, BC_3_ and BC_4_ generations, respectively (Table 3).

**Table 3:** Segment transmission rates from F_1_ to BC_1_ through to BC_4_ generations of both the A^t^ and G genomes of *T. timopheevii* in the hexaploid wheat background

Linkage group	Genome	In 35 BC_1_ plants	In 134 BC_2_ plants	In 144 BC_3_ plants	In 33 BC_4_ plants
		(%)	(%)	(%)	(%)
LG1	At	25	67	41	7
		(71.4)	(50)	(28.5)	(21.2)
	G	14	26	10	0
		(40)	(19.4)	(6.9)	(0)
LG2	At	28	69	52	4
		(80)	(51.5)	(36.1)	(12.1)
	G	33	101	107	23
		(94.3)	(75.4)	(74.3)	(69.7)
LG3	At	26	72	58	4
		(74.3)	(53.7)	(40.3)	(12.1)
	G	23	48	24	0
		(65.7)	(35.8)	(16.7)	(0)
LG4	At	25	51	34	5
		(71.4)	(38.1)	(23.6)	(15.2)
	G	30	69	41	6
		(85.6)	(51.1)	(28.5)	(18.2)
LG5	At	33	90	71	8
		(94.3)	(67.2)	(49.3)	(24.2)
	G	32	76	40	3
		(91.4)	(56.7)	(27.8)	(9.1)
LG6	At	30	68	43	11
		(85.7)	(50.8)	(29.9)	(33.3)
	G	27	58	43	9
		(77.1)	(43.3)	(29.9)	(27.3)
LG7	At	29	75	49	6
		(82.9)	(56)	(34)	(18.2)
	G	25	50	42	7
		(71.4)	(37.3)	(29.2)	(21.2)

### Fluorescence in situ hybridisation (FISH) validates the molecular markers

**Fig. 4:**
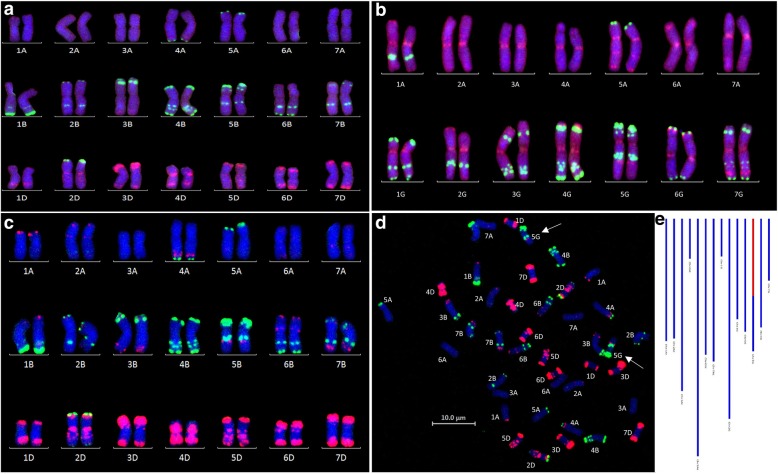
FISH validation of the SNP genotyping of wheat-*T. timopheevii* introgression lines. Oligos pSc119.2–1 (green) and the Afa family (red) were used as FISH probes. **a** known FISH karyotype of wheat [56] (**b**) FISH karyotype of *T. timopheevii* accession P95–99.1-1 (based on that published by [37]) (**c**) FISH karyotype of line BC_3_F_2_–114-1 showing a homozygous introgression from *T. timopheevii* linkage group 5G into wheat chromosome 5B (**d**) FISH signals in the metaphase spread of line BC_3_F_2_–114-1 used to make the karyotype with white arrows indicating chromosomes T5GS.5GL-5BL (**e**) GGT bar diagram of line BC_3_F_2_–114-1 showing the *T. timopheevii* introgression from linkage group 5G in red markers while the wheat alleles are represented in blue

To confirm the presence of segments indicated by the genotyping, FISH was carried out on selected individuals with large *T. timopheevii* segments using oligos pSc119.2–1 [36] and the Afa family [39]. Firstly, it was confirmed that the FISH karyotype of the *T. timopheevii* accession used in this work was similar to a previously published karyotype for *T. timopheevii* (Fig. 4b; [37]). Differences between the FISH signals of wheat (Fig. 4a; [56]) and *T. timopheevii* were subsequently used to identify *T. timopheevii* segments in a wheat background (Figs. 4c-d) and validate the genotyping by molecular markers (Fig. 4e). While it was not possible to detect all the segments carried by an individual plant using this technique, either due to small size or no visible difference between the FISH signals for wheat and *T. timopheevii*, it was possible to confirm the expected larger segments.

### Comparative analysis between *T. timopheevii* and wheat genomes

Synteny analyses were carried out separately for both the A^t^ and G genomes with wheat using sequence information of the markers located on the genetic map of *T. timopheevii*. The sequences of the mapped markers were compared using BLAST (e-value cut-off of 1e-05) against the wheat genome sequence (Refseq v1; [20]) to obtain orthologous map positions of the best BLAST hits in the A, B and D genomes of wheat. 77.9% of the markers on the A^t^ genome had an overall top hit on the A genome of wheat and 72.2% of the markers on the G genome had an overall top hit on the B genome of wheat. This result was expected since the A^t^ and A genomes are closely related through a common progenitor *T. urartu* [11] and the G and B genomes are also thought to be closely related [12, 44].

**Fig. 5:**
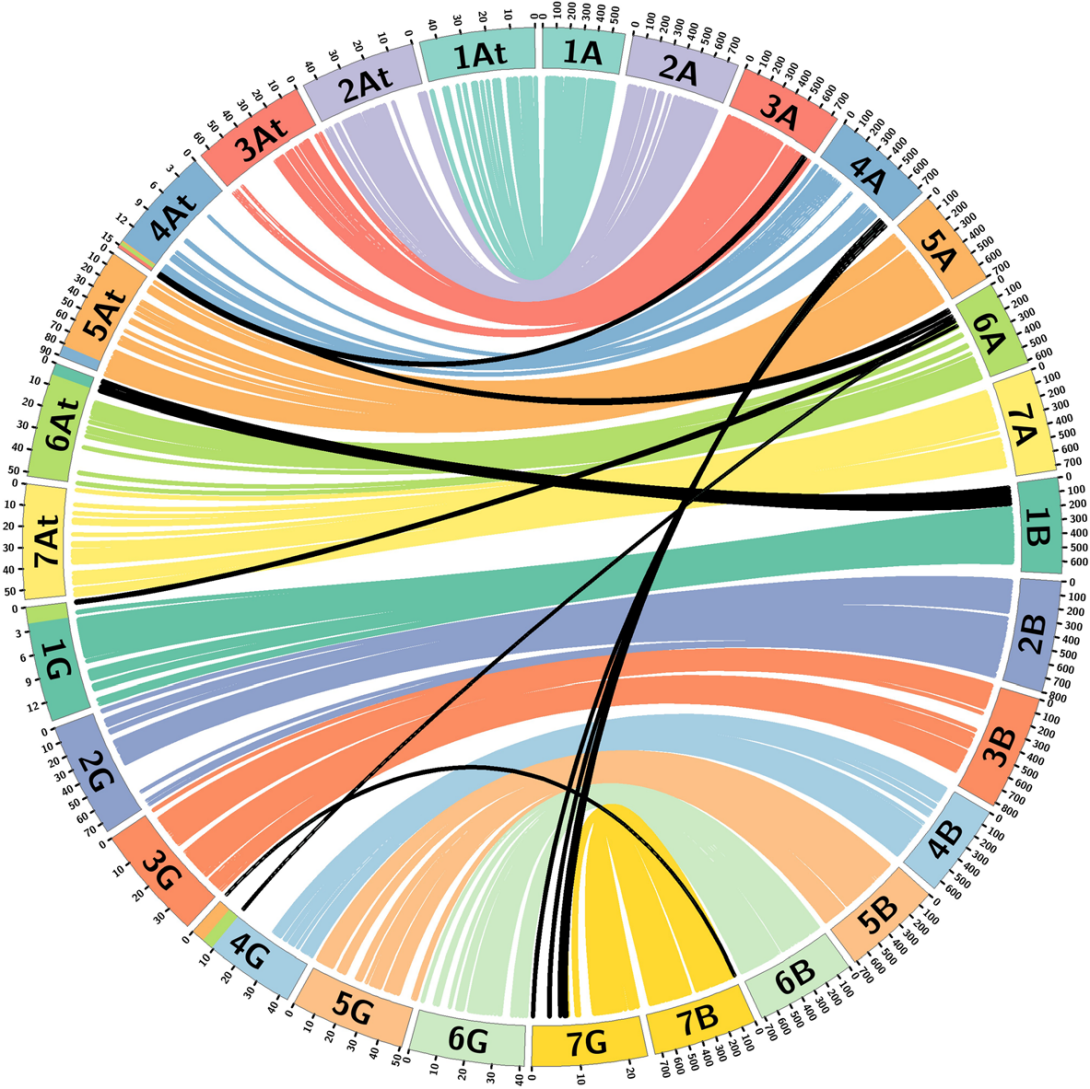
Comparison of the A^t^ and G genomes of *T. timopheevii* and the A and B genomes of wheat, respectively, showing significant synteny and inter- and intra-genomic translocations. Chromosomes from both genome groups (A^t^/A and G/B) are represented by differently coloured ideograms but chromosomes from the same homoeologous group in *T. timopheevii* and wheat are represented by the same colour. Ticks on the *T. timopheevii* genomes show the ideogram size in cM whereas those on the wheat genomes show the ideogram size in Mbp. BLAST results are represented by differently coloured links between the map positions of the markers on the genetic map of *T. timopheevii* and their corresponding physical positions on the wheat genome. Syntenic links are of the same colour as the homoeologous chromosomes which are linked whereas black links indicate where the BLAST hit was to a non-homoeologous chromosome

**Fig. 6:**
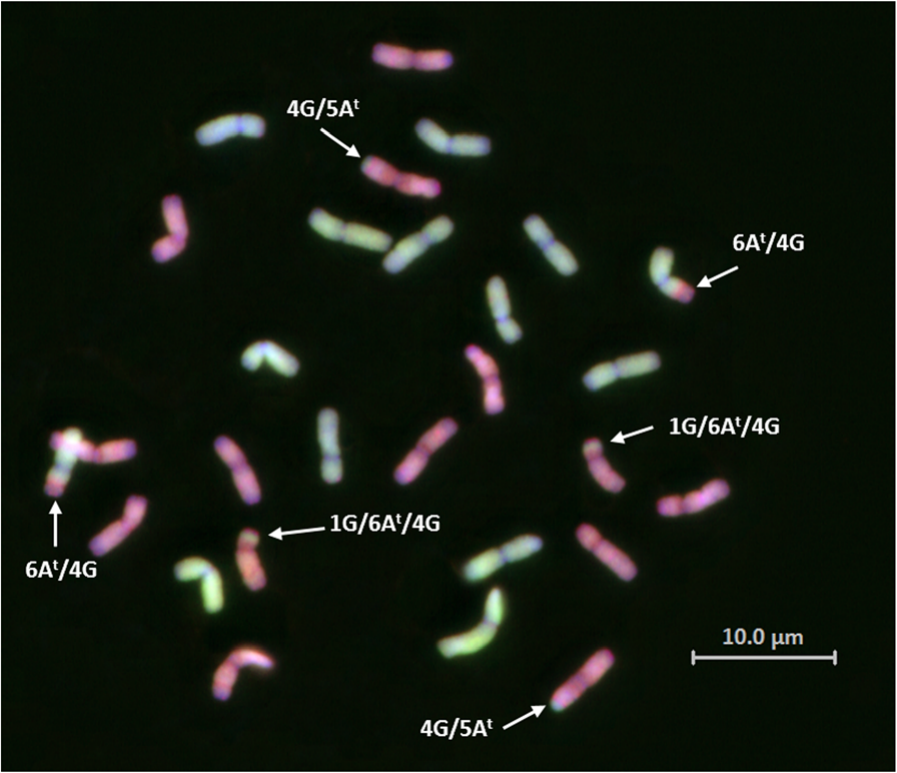
Multi-colour GISH of a metaphase spread of *T. timopheevii* accession P95–99.1-1. Chromosomes of the A^t^ genome are shown in green and chromosomes of the G genome in red. White arrows indicate the inter-genomic translocations 6A^t^S/1GS/4GS and 4GS/4A^t^L (the intra-genomic translocation 4A^t^L/3A^t^Lcould not be detected using multi-colour GISH)

The synteny analysis in Fig. 5 shows the comparison of the A^t^ and G genomes of *T. timopheevii* with the A and B genomes of wheat respectively. Figure 5 shows different coloured links, corresponding to the colour of the ideograms between *T. timopheevii* and wheat, which represent the physical positions of the markers on the wheat genome chromosomes as obtained through BLAST analysis. These results show that macro-synteny and collinearity is maintained between the A^t^ and G genomes of *T. timopheevii* and the A and B genomes of wheat respectively. Black links show where the BLAST hit of the marker sequence is on a non-homoeologous chromosome in wheat potentially indicating that there are chromosomal translocations within *T. timopheevii*. All chromosomal translocations are also shown as highlighted chromosome ideograms in the A^t^ and G genomes of *T. timopheevii* with part of the chromosome involved in the translocation highlighted with the colour of the chromosome that has been translocated onto it. Some of the inter-genome chromosomal translocations include 1GS/6A^t^S, 4GS/6A^t^S and 4GS/5A^t^L. The intra-genome chromosomal translocations include 4A^t^L/3A^t^L, 4A^t^L/6A^t^S and 4A^t^L/5A^t^L (the latter not indicated by a black link since this translocation is already present in wheat). Translocation 4A^t^L/5A^t^L, which the emmer wheat inherited from *T. urartu* [10, 40, 41] has been previously reported in *T. timopheevii* [23]. All the other translocations are potentially species-specific and have also been previously reported [22, 33] as having occurred potentially through three translocation events namely 6A^t^S/1GS/4GS, 4GS/4A^t^L, and 4A^t^L/3A^t^L, which most likely arose in that sequence. The 4A^t^L/5A^t^L translocation has been previously reported in wheat as part of a double translocation 5AL/4AL/7BS [32, 41], however, Fig. 5 shows that the 4AL/7BS translocation (or 4A^t^L/7GS in this case) does not exist in *T. timopheevii* matching previous reports [33]. These results also confirm previous reports that chromosomes 1A^t^, 2A^t^, 5A^t^, 7A^t^, 2G, 3G, 5G, and 6G of *T. timopheevii* do not differ structurally from their counterpart in the A and B genomes of wheat [2, 33]. Figure 6 shows a multi-colour GISH image of a metaphase spread showing some of these translocations.

## Discussion

*T. timopheevii* is a potentially important source of genetic variation for a range of agronomically important traits (see Introduction). In the past, one of the critical bottlenecks limiting the use of genetic variation from the wild relatives of wheat for crop improvement has been the inability to rapidly detect and characterise introgressions. However, the use of an Axiom® SNP genotyping array [18, 19, 27, 28], in combination with FISH, has led to the identification of 276 putative wheat-*T. timopheevii* introgressions.

The F_1_ interspecific hybrids generated between Paragon and *T. timopheevii*, which were backcrossed to Paragon (Fig. 1), were essentially haploid for the A, B and D genomes of wheat and also the A^t^ and G genomes of *T. timopheevii*. Therefore, intraspecific recombination between homologous chromosomes during meiosis was not possible. Thus, the rationale for using the strategy employed was to attempt to increase the frequency of recombination between 1) the A genome of wheat and the A^t^ genome of *T. timopheevii* and 2) the B genome of wheat and the G genome of *T. timopheevii*.

It has previously been shown that the A and A^t^ genomes and the B and the G genomes recombine, at a frequency of 70 and 30% respectively, in the presence of the *Ph1* locus located on the long arm of chromosome 5B of wheat [14] (*T. timopheevii* also carries a pairing control locus/gene although it has been reported to have a weaker affect than *Ph1* in wheat, [45]). This chromosome pairing study indicates that the A and B genomes of wheat do not show complete homology with the A^t^ and G genomes of *T. timopheevii*. In an attempt to increase recombination between the genomes of wheat and *T. timopheevii*, a *ph 1* mutant wheat was used to generate F_1_ hybrids, i.e. the resulting interspecific hybrids thus lacked the 5B *Ph1* locus but carried its weaker putative *T. timopheevii* allelic variant [45]. Since a control experiment was not undertaken as this was not the object of this research (i.e. the object of this research was purely to generate large numbers of introgressions for exploitation in breeding programmes) it was not possible to determine if removal of the 5B *Ph1* locus increased recombination between wheat and *T. timopheevii* chromosomes. However, we note that significant numbers of wheat/*T. timopheevii* introgressions, i.e. 276, were generated. The fact that some linkage groups of *T. timopheevii,* derived from introgressions, had much lower frequencies of recombination than others, e.g. for the G genome 16.6 cM for linkage group 1 in contrast to linkage group 2 which was 76.5 cM (Fig. 2 and Table 2), indicates that the level of homology between these two species varies across the genome.

Large numbers of wheat/wild relative introgressions were also observed in recent work on *Am. muticum* (218 – [27]), *Ae. speltoides* (294 – [28]) and *T. urartu* (176 – [19]). In contrast, the numbers of introgressions obtained between wheat and *Th. bessarabicum* was extremely low, i.e. only 12 [18]. The lower frequency of wheat/*Th. bessarabicum* introgression observed may result from the fact there was considerable disruption in synteny between this species and the genomes of wheat as compared to the other species described.

The F_1_ hybrids contained three genomes from wheat (A, B and D) and two genomes from *T. timopheevii* (A^t^ and G). Thus, even though a degree of recombination was expected to occur between the A/A^t^ and B/G genomes, it was expected that a large proportion of the gametes produced would be unbalanced. As a result, it was predicted that the fertility of the F_1_ hybrids would be low. This was found to be the case with only 16% of crossed F_1_ hybrid ears setting seed (Table 1). A very similar level of infertility was observed in F_1_ hybrids between wheat and *Am. muticum* (A, B, D and T) [27]. However, wheat/*Ae. speltoides* F_1_ hybrids (A, B, D and S) exhibit higher fertility, i.e. 29% [28]. As a result of the infertility of the wheat/T*. timopheevii* F_1_ hybrids, only 152 BC_1_ seed were generated from 351 crossed ears with Paragon. Twenty-five F_1_ seeds gave rise to the BC_1_, BC_2_, BC_3_ and BC_4_ populations in this work. The level of interspecific recombination detected by genetic mapping was such that it was possible to assemble 14 linkage groups of *T. timopheevii*. Furthermore, the use of blast analysis enabled us to determine which linkage groups were derived from which two genomes of *T. timopheevii*. We were able to characterise these introgressions and track them through the backcross generations (Fig. 3). However, while we used the genetic map to identify and characterise introgressions, it is important to note that the maps were not produced using proper mapping families and thus the cM distances should be treated with caution.

All of the backcross populations were derived from gametes of 25 F_1_ plants. Recombination between the chromosomes of wheat and *T. timopheevii* was not limited to the gametes of the F_1_ population, i.e. genotyping showed that interspecific chromosome recombination had occurred in the gametes of individuals of the backcross populations in the presence of the wild type *Ph1* locus. However, SNP analysis from each of the backcross generations showed that the majority occurred due to recombinant events in the F_1_ gametes rather than in later generations (Fig. 3).

In total, out of 276 introgressions, 141 involved the A^t^ genome and 135 involved the G genome of *T. timopheevii*. Slightly more A^t^ introgressions were observed than G introgressions. The difference in the number of introgressions involving the A^t^ chromosomes may result from a higher frequency of recombination of this genome with the genomes of wheat (presumably the A genome). However, the difference in the number of introgressions could reflect marker coverage, i.e. there may be insufficient markers to accurately access the actual levels of recombination occurring with each of the *T. timopheevii* genomes with those of wheat. Thus, it is therefore not possible to reliably compare and contrast the frequencies of recombination of the A^t^ and G genomes of *T. timopheevii* with wheat in this work.

Validation of introgressions identified via the Axiom® array was performed on selected individuals, potentially carrying large *T. timopheevii* segments, using FISH. Unlike GISH [27, 28], FISH could only be used to detect introgressions when the resulting hybridisation signals of oligos pSc119.2–1 and the Afa family could distinguish wheat chromosomes from *T. timopheevii* chromosomes. However, in each case where FISH analysis was undertaken it confirmed the SNP analysis (Fig. 4).

In this work it was found that chromosome 2G was transmitted at a much higher frequency to each of the backcross generations than other chromosomes from *T. timopheevii* (Table 3). This work confirms similar findings for the elevated transmission of either a complete chromosome 2G [42, 57] or a 2G introgression in wheat [9]. Chromosome 2S from *Ae. speltoides* has also been shown to carry a gametocidal gene which results in its preferential transmission to the next generation through both the male and female gametes [28, 34, 35, 58]. However, while the level of preferential transmission exhibited by chromosome 2S is at, or close to, 100% [28, 34], this was not found to be the case with chromosome 2G. Thus, the gene(s) responsible for the preferential transmission of chromosome 2G from *T. timopheevii* appear to be weaker than the gene for preferential transmission on chromosome 2S from *Ae. speltoides*.

One of the implications of preferentially transmitted chromosomes is that they need to be removed if introgressions without them are to be developed. In the case of chromosome 2S from *Ae. speltoides* this is extremely difficult as it is transmitted exclusively to the next generation. Removal of these genes thus requires the use of additional strategies [15]. However, since chromosome 2G is not transmitted exclusively, it can be removed via further backcrossing combined with selection, e.g. molecular markers, FISH.

One of the major bottle-necks preventing the exploitation of the vast reservoirs of genetic variation in the wild relatives for wheat improvement has been the lack of a high-throughput system to detect and characterise introgressions. The Axiom® Wheat-Relative Genotyping Array was selected as the technology to identify wheat/wild relative introgressions for the work being undertaken on multiple species at the Nottingham BBSRC Wheat Research Centre. A key factor in choosing this technology was that a single array is able to identify introgressions from all of the ten species being analysed in the programme within the wheat genotypes being used. To date it has been used to identify large numbers of introgressions into wheat from *Am. muticum*, *Ae. speltoides*, *Th. bessarabicum*, *T. urartu* and *Th. intermedium* ([27] and 2018; [19] and b; [8]).

While the Axiom® Wheat-Relative Genotyping Array has proved invaluable in detecting and characterising introgressions, in our hands, we were only able to utilise 1582 of the markers to identify wheat/*T. timopheevii introgressions*. However, it should be noted that since the 35 K SNPs on the array were selected to be polymorphic with all ten wild relatives used in the programme, only a subset of the SNPs (~ 19.5 K) were polymorphic with *T. timopheevii* to begin with. In addition, for a majority of the polymorphic SNPs (~ 10.5 K), at least one parent showed a heterozygous call. Therefore, a smaller group of markers showing a typical cluster pattern for a codominant marker were selected for genetic mapping.

In addition to the array, other methods for the detection of wheat/wild relative introgressions could be exploited in the future, including next generation sequencing technologies such as genotyping by sequencing (GBS; [53]) and Specific Locus Amplified Fragments (SLAF-seq; [55]). Population-specific SNPs can be discovered through the GBS procedure through sequencing of DNA libraries obtained after restriction digest of samples. Unlike the Axiom array, GBS is free of ascertainment bias [50] but the utility of GBS for this type of work in the future will depend on the cost of sequencing, bioinformatics, etc. SLAF-seq is an alternative method that could be used to identify introgressions. However, as many of the SNPs generated by this technology are likely to be from non-genic regions, only a limited number are likely to be transferable between species. Thus, it could be necessary to undertake the SLAF-seq protocol for each of the wild relatives and the wheat genotype in each introgression programme. Whichever technologies are used, they will need to take into account that some of the wild relatives are out-breeders and some are polyploids (i.e. the technologies will need to be able to distinguish polymorphisms that are between a wild relative and wheat versus polymorphisms between alleles within a wild relative).

Irrespective of the detection platform used the technologies already available, and those that will become available in the future, are resulting in a step change in our ability to detect wheat/wild relative introgressions that will allow us, for the first time, to begin to systematically exploit the vast reserve of genetic variation available in the wild relatives for wheat improvement.

In the present work we are currently unable to use SNP markers on the Axiom® array or FISH to determine which chromosomes of wheat are involved in each of the introgressions generated in this work. However, we are developing wheat chromosome-specific Kompetitive Allele Specific PCR (KASP™) markers which allow the high- throughput analysis of large numbers of introgressions enabling us to track introgressions in future derivative material and also to determine which wheat chromosomes have introgressions from *T. timopheevii*.

## Conclusions

In this work, we have used the Axiom® Wheat-Relative Genotyping Array for high-throughput genotyping of wheat-*T. timopheevii* introgression lines. The characterisation of these interspecific hybrid lines has resulted in the development of a set of SNP markers, spread across all 7 chromosomes of both the A^t^ and G genomes, that can detect the presence of *T. timopheevii* in a hexaploid wheat background making it a potentially valuable tool for marker assisted selection (MAS) in wheat pre-breeding programs. These valuable resources of high-density molecular markers and wheat-*T. timopheevii* hybrid lines will greatly enhance the work being undertaken for wheat improvement through wild relative introgressions.

## Methods

### Generation of introgressions

It has previously been shown that even in the presence of the *Ph1* locus, the A^t^ and G genomes of *T. timopheevii* are sufficiently closely related to the A and B genomes of wheat for a level of recombination, and hence genetic exchange, to occur at meiosis in hybrids between the two species, with the resulting generation of interspecific recombinant chromosomes or introgressions. In order to generate introgressions, hexaploid wheat cv. Paragon *ph 1/ph1* mutant (2n = 2x = 14) (obtained from the Germplasm Resource Unit (GRU) at the John Innes Centre) was pollinated with *T. timopheevii*, (accession P95–99.1-1, obtained from the United States Department of Agriculture, USDA; 2n = 4x = 28), to produce F_1_ interspecific hybrids. These hybrids were then grown to maturity and backcrossed as the female parent with Paragon to generate BC_1_ populations. The BC_1_ individuals and their resulting progenies were recurrently pollinated to produce BC_2_, BC_3_ and BC_4_ populations (Fig. 1).

### Genotyping of introgression lines

A 35 K Axiom® Wheat-Relative Genotyping Array (Affymetrix, Santa Clara, California) was used to detect the presence of putative wheat/*T. timopheevii* introgressions in each of the backcross generations [18, 19, 27, 28]. This array is composed of SNPs between various wild relatives and wheat genotypes, including *T. timopheevii* [27]. All the SNPs incorporated in this array formed part of the Axiom® 820 K SNP array [66], the data-set for which is available from www.cerealsdb.uk.net [64, 65]. Table 2 shows the number of putative SNPs between *T. timopheevii* and each of the wheat genotypes included on the array. The array allows 384 lines to be screened at one time. Genotyping was performed as described by King et al. [27] with slight modifications (see below).

DNA was extracted according to the Somers and Chao protocol (http://maswheat.ucdavis.edu/PDF/DNA0003.pdf, verified 21 January 2019, original reference in [46]) from 344 individuals of the back-crossed populations, BC_1_, BC_2_, BC_3_, and BC_4_, derived from the wheat/*T. timopheevii* F_1_ hybrids and control samples which included three replicates of each of the parental lines, i.e. wheat cv. Paragon and *T. timopheevii*. These populations were genotyped with the Axiom® Wheat-Relative Genotyping Array. Only Poly High Resolution (PHR) SNP markers, which were co-dominant and polymorphic, with at least two examples of the minor allele were used for genetic mapping [27]. Call rate for a sample was calculated as the percentage of the number of SNP probes on the array that resulted in a definitive genotype call (AA, AB, BB) for that sample. The equipment, software, procedures and criteria used for this genotyping are as described by King et al. [27].

### Genetic mapping

SNP markers which showed: 1) heterozygous calls for either parent(s) 2) no polymorphism between the wheat parents and *T. timopheevii* and/or 3) no calls for either parent(s) were removed using Flapjack™ ([38]; v.1.14.09.24). The resulting markers were sorted into linkage groups (Fig. 2) in JoinMap® 4.0 [61] with a LOD score of 50. All markers that did not show any heterozygous calls or were unlinked were ignored and only the highest-ranking linkage groups with more than 30 markers were selected for map construction. Linkage groups were assigned to one of the 14 chromosomes of *T. timopheevii* through a BLAST analysis against the wheat genome reference sequence (RefSeq v1.0; [20]). Markers from each linkage group were used in a BLAST function (e-value cut-off of 1e-05) against the wheat genome to obtain the orthologous map positions of the top hits in the A, B and D genomes of wheat. If majority of the markers from a linkage group had a top hit in the A genome of wheat, they were assigned to the A^t^ genome of *T. timopheevii*. Similarly, if most markers from a linkage group had a top hit on the B genome of wheat then the group was mapped to the G genome of *T. timopheevii*. The linkage groups were assigned to the same homoeologous group in *T. timopheevii* as indicated by the BLAST results in wheat. Linkage group data was used to produce two genetic maps (one each for the A^t^ and the G genome of *T. timopheevii* – Fig. 2a and b) using MapChart 2.3 [62]. Markers at the same genetic map position were ordered according to their physical positions on the wheat genome (RefSeq v1.0; [20]) but only the first two markers were selected as anchors and represented on the map for that position. All markers and their order in the linkage group are shown in Additional file 1 Graphical genotype visualization was performed using Graphical GenoTypes 2.0 (GGT; [60]).

### Cytogenetic analysis

#### Preparation of metaphase spreads

Preparation of chromosome spreads was as described in Zhang et al. [68] and King et al. [27] but briefly: Roots were excised from germinated seeds, treated with nitrous oxide gas at 10 bar for 2 h, fixed in 90% acetic acid for 10 min and then washed three times in water on ice. Root tips were dissected and digested in 20 μl of 1% pectolyase Y23 and 2% cellulase Onozuka R-10 (Yakult Pharmaceutical, Tokyo) solution for 50 min at 37 °C and then washed three times in 70% ethanol. Root tips were crushed in 70% ethanol, cells collected by centrifugation at 5000 rpm for 1 min, briefly dried and then re-suspended in 30–40 μl of 100% acetic acid prior to being placed on ice. The cell suspension was dropped onto glass slides (6–7 μl per slide) in a moist box and dried slowly under cover.

#### Fluorescence in situ hybridisation (FISH)

Slides were initially probed for multi-colour fluorescence in situ hybridization (FISH). Two repetitive DNA sequences pSc119.2 [36] and the Afa family [39] were labelled with Alexa Fluor 488–5-dUTP and Alexa Fluor 594–5-dUTP, respectively, and hybridized to the slides.

#### Genomic in situ hybridization (GISH)

The protocol for GISH was as described in Zhang et al. [68], Kato et al. [25] and King et al. [27]. Genomic DNAs was isolated from *T. urartu* (A genome) and *Ae. speltoides* (B genome) and labelled by nick translation with Chroma Tide Alexa Fluor 488–5-dUTP (Invitrogen, Carlsbad, California; C11400) and Alexa Fluor 594–5-dUTP (Invitrogen; C11397), respectively.

Slides of *T. timopheevii* (accession P95–99.1-1) were probed with labelled DNAs of *T. urartu* (100 ng) and *Ae. speltoides* (200 ng) in a ratio of 1:2 per slide to detect the A^t^A^t^GG genomes. Slides were counterstained with Vectashield mounting medium with DAPI, and analysed using a Zeiss Axio ImagerZ2 upright epifluorescence microscope (Carl Zeiss Ltd., Oberkochen, Germany) with filters for Alexa Fluor 488 and Alexa Fluor 594. Photographs were taken using a MetaSystems Coolcube 1 m CCD camera. Image analysis was carried out using Meta Systems ISIS and Metafer software (Metasystems GmbH, Altlussheim, Germany).

### Comparative analysis

Synteny analysis was carried out using sequence information of the markers located on the genetic map of *T. timopheevii*. The sequences of the mapped markers were compared using BLAST (e-value cut-off of 1e-05) against the wheat genome reference sequence (RefSeq v1.0; [20]) to obtain the orthologous map positions of the top hits in the A, B and D genomes of wheat (Fig. 5). To generate the figures, cM distances on the linkage groups of the present map of *T. timopheevii* were scaled up by a factor of 100,000 to match similar base pair lengths of the chromosomes of the wheat genome. Figure 5 was visualized using Circos (v. 0.67; [30]) with chromosomes from both genome groups (A^t^/A and G/B) being represented by differently coloured ideograms. Ideograms from the same homoeologous group between *T. timopheeevii* and wheat are represented by the same colour, e.g. chromosomes 1A^t^ and 1A are represented by the same colour. Corresponding genetic and physical positions of the markers on *T. timopheevii* and wheat, respectively, are shown in Additional file 1.

## Additional file

Additional file 1: Physical positions on the A, B and D genomes of wheat of all markers present on the genetic maps of the A^t^ and G genomes of *T. timopheevii*, as obtained through BLAST against the wheat genome (RefSeq v1; [20]). Positions highlighted in yellow were the top hits in the BLAST search and markers highlighted in green represent a translocated region of the *T. timopheevii* chromosomes (XLSX 98 kb)

## Abbreviations

BLAST: basic local alignment search tool
FISH: fluorescence in situ hybridisation
GISH: genomic in situ hybridisation
PHR: poly high resolution
SNP: single nucleotide polymorphism
